# Identifying pregnancy episodes and estimating the last menstrual period using an administrative database in Korea: an application to patients with systemic lupus erythematosus

**DOI:** 10.4178/epih.e2024012

**Published:** 2023-12-19

**Authors:** Yu-Seon Jung, Yeo-Jin Song, Jihyun Keum, Ju Won Lee, Eun Jin Jang, Soo-Kyung Cho, Yoon-Kyoung Sung, Sun-Young Jung

**Affiliations:** 1 Chung-Ang University College of Pharmacy, Seoul, Korea; 2 Department of Rheumatology, Hanyang University Hospital for Rheumatic Diseases, Seoul, Korea; 3 Hanyang University Institute for Rheumatology Research, Seoul, Korea; 4 Department of Obstetrics and Gynecology, Hanyang University College of Medicine, Seoul, Korea; 5 Department of Global Innovative Drugs, Graduate School of Chung-Ang University, Seoul, Korea; 6 Department of Information Statistics, Andong National University, Andong, Korea

**Keywords:** Algorithms, Pregnancy, Pregnancy outcome, Administrative claims healthcare, Immunosuppressive agents, Lupus erythematosus systemic

## Abstract

**OBJECTIVES:**

This study developed an algorithm for identifying pregnancy episodes and estimating the last menstrual period (LMP) in an administrative claims database and applied it to investigate the use of pregnancy-incompatible immunosuppressants among pregnant women with systemic lupus erythematosus (SLE).

**METHODS:**

An algorithm was developed and applied to a nationwide claims database in Korea. Pregnancy episodes were identified using a hierarchy of pregnancy outcomes and clinically plausible periods for subsequent episodes. The LMP was estimated using preterm delivery, sonography, and abortion procedure codes. Otherwise, outcome-specific estimates were applied, assigning a fixed gestational age to the corresponding pregnancy outcome. The algorithm was used to examine the prevalence of pregnancies and utilization of pregnancy-incompatible immunosuppressants (cyclophosphamide [CYC]/mycophenolate mofetil [MMF]/methotrexate [MTX]) and non-steroidal anti-inflammatory drugs (NSAIDs) during pregnancy in SLE patients.

**RESULTS:**

The pregnancy outcomes identified in SLE patients included live births (67%), stillbirths (2%), and abortions (31%). The LMP was mostly estimated with outcome-specific estimates for full-term births (92.3%) and using sonography procedure codes (54.7%) and preterm delivery diagnosis codes (37.9%) for preterm births. The use of CYC/MMF/MTX decreased from 7.6% during preconception to 0.2% at the end of pregnancy. CYC/MMF/MTX use was observed in 3.6% of women within 3 months preconception and 2.5% during 0-7 weeks of pregnancy.

**CONCLUSIONS:**

This study presents the first pregnancy algorithm using a Korean administrative claims database. Although further validation is necessary, this study provides a foundation for evaluating the safety of medications during pregnancy using secondary databases in Korea, especially for rare diseases.

## GRAPHICAL ABSTRACT

[Fig f3-epih-46-e2024012]

## Key Message

Limited safety data for pregnant women prompted recent studies on medication during pregnancy using real-world databases. This study developed a tailored algorithm for Korean healthcare claims database, employing a hierarchy of pregnancy outcomes and incorporating pre-term delivery and sonography codes for last menstrual period estimation. Applied to systemic lupus erythematosus (SLE) patients, this study presented the prevalence and drug utilization pattern of pregnancy-incompatible immunosuppressants from preconception to pregnancy end, laying a foundation for further claims database studies on medication pregnancy safety.

## INTRODUCTION

Pregnant women represent a unique population typically not included in clinical trials, and most clinical decisions regarding medication use during pregnancy are based on animal studies, case reports, and a few post-surveillance studies using registry data. Considering the scarcity of safety data with respect to pregnant women, recent studies have attempted to examine medication safety during pregnancy using routinely compiled administrative claims databases. Administrative claims databases offer several advantages, including large sample sizes with relatively low costs and no recall bias; however, misclassification of pregnancy outcomes and the last menstrual period (LMP) due to missing information, miscoding, and cross-coding is a common disadvantage [1,2].

In several countries, pregnancy algorithms based on administrative claims databases have been developed and adapted according to data availability, clinical practice patterns, and the healthcare system to minimize the misclassification of pregnancy outcomes and the LMP [3-9]. The agreement, expressed as percentage, between pregnancy outcomes identified by algorithms and reviewer decisions is 96-100% for live births, 70.8-100% for stillbirths, and 92-100% for spontaneous abortions [4,6,9,10]. Despite the validity of published algorithms, they are not directly applicable to the Korean national claims database, and a new algorithm considering code availability and practices in Korea needs to be proposed.

Pregnancy studies using claims databases in Korea often only involve live births, which can be linked to infant claims, and identify the LMP by subtracting a fixed number of weeks, which is usually the average gestational age (GA) for corresponding pregnancy outcomes [11-13]. The associated algorithms can have high validity for pregnancy outcomes while overlooking pregnancy loss and teratogenic effects resulting in pregnancy loss [14]. Consecutive pregnancy episodes of an individual also cannot be fully captured, and overlapping episodes are not considered. Refining the pregnancy algorithm would further reduce the misclassification bias of medication exposure during preconception and certain periods of pregnancy (first, second, and third trimesters). Therefore, developing a pregnancy algorithm using a nationwide claims database in Korea would facilitate further investigation of the safety of medication use during pregnancy, considering the timing of exposure and including outcomes with pregnancy loss.

The objectives of our study were (1) to develop an algorithm for identifying pregnancy episodes and estimating the LMP using a Korean claims database and (2) to examine pregnancy outcomes in patients with systemic lupus erythematosus (SLE) and the use of pregnancy-incompatible immunosuppressants before and during pregnancy. SLE is an autoimmune disease predominant in women of childbearing age, and patients with SLE have a higher incidence of adverse pregnancy outcomes than observed in the general population [15,16]. Women with SLE who are considering pregnancy are advised to discontinue pregnancy-incompatible immunosuppressants for up to 3 months before the LMP [15,17]. However, although pre-family planning is recommended, it is not universally implemented in clinical practice [18]. Therefore, investigating the prevalence of pregnancy-incompatible drug use in clinical settings is important, as it may play a role in the higher risk of adverse pregnancy outcomes in patients with SLE.

## MATERIALS AND METHODS

### Data sources

We used nationwide healthcare claims data from the National Health Information Database (NHID) of Korea, which provides all citizens’ eligibility data (income-based insurance contributions, demographic variables, and date of death), national screening data, and healthcare utilization data (inpatient and outpatient usage and prescription records) [19].

### Algorithm

The study algorithm used codes from the Korean Standard Classification of Diseases, seventh revision (KCD-7), which are based on the International Classification of Diseases, 10th revision (ICD-10), and procedure codes for claims data from 2002 to 2018. It involved following two steps: (1) determination of pregnancy outcomes (delivery, stillbirth, and abortion) and (2) estimation of the LMP. Women were allowed multiple pregnancies, and all episodes indicative of pregnancy in women of childbearing age (12-49 years) at the time of the pregnancy outcome were included.

### Determination of pregnancy outcomes

Following pregnancy outcomes were defined using KCD-7 or procedure codes based on pregnancy studies in Korea: delivery (full-term, preterm), stillbirth, and abortion (spontaneous, induced) [12,20-22]. A list of codes defining each outcome is provided in Supplementary Material 1. An obstetrician-gynecologist confirmed definition using KCD-7 codes for stillbirth and abortion and procedure codes for delivery in clinical practice. During our pre-analysis of the NHID, we found that procedures for delivery were recorded in 99.4% of potential delivery claims, indicating delivery by either KCD-7 or procedure codes (Supplementary Material 2). KCD-7 codes for abortion were present in 97.5% of potential abortion claims by either KCD-7 or procedure codes (Supplementary Material 3). Therefore, we chose to use procedure codes for delivery, as they are commonly recorded for reimbursement purposes, whereas for abortions, we opted for diagnosis codes, as not all abortions require medical procedures.

**Figure 1.**
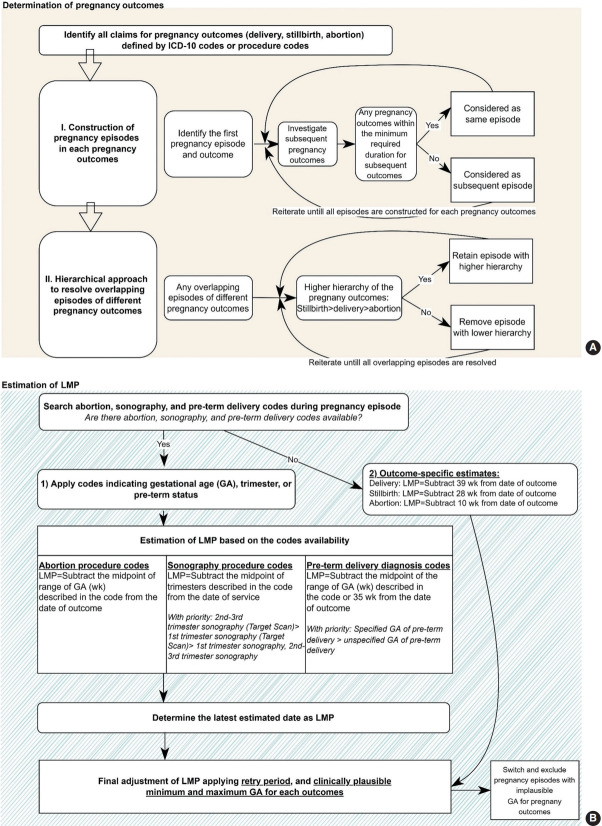
Algorithms (A) determining pregnancy outcomes, and (B) estimating last menstrual period (LMP) from the claims database. ICD-10, International Classification of Diseases, 10th revision.

The clinically plausible duration required for subsequent episodes and a hierarchical approach were adapted from a published pregnancy algorithm to identify true episodes from multiple claims (Figure 1A) [4-6,10]. Pregnancy episodes were defined as the period between the estimated date of the LMP and pregnancy outcome. As illustrated in Supplementary Material 4, the first claim of pregnancy-related codes within the available timeframe of an individual patient was assigned as the first pregnancy episode. Multiple claims within the clinically plausible duration for subsequent episodes were considered to be part of the same episode, and claims outside the minimum duration were categorized as subsequent episodes (Supplementary Material 5). The step was repeated for all pregnancy outcomes. Subsequently, a hierarchical approach was applied to resolve overlapping episodes. Pregnancy episodes with a lower hierarchy were considered miscoded and were eliminated.

Considering the validity of outcomes, the hierarchy of pregnancy outcomes was adapted from pregnancy studies using Korean claims databases [4-6,10]. A study using a claims database with a mother–infant link in Korea confirmed that 95.3% of all deliveries were linked to the infant record, which has shown high validity of the delivery code [20]. Stillbirth is considered a higher priority than delivery because it can coexist with delivery claims. Therefore, the following hierarchy of pregnancy outcomes was used in our study: stillbirth> delivery> abortion.

### Estimation of the menstrual period

We estimated the LMP using two distinct methods, depending on the presence of procedures for abortion, sonography, and diagnosis for preterm delivery within each episode, as described in Figure 1B [3-5].

First, when abortion or sonography procedures or preterm delivery diagnoses were present, we estimated the LMP by subtracting the midpoint of the specified trimester or GA from the outcome date [6] (Supplementary Material 6-1). To ensure clinical validity, a hierarchy was applied based on reimbursement guidelines, verified by obstetrician-gynecologists, and codes with a narrower range of the indicated GA or trimester had higher priority. In cases of conflicting information on GA or trimesters on same date, the lowest GA was chosen. Priority among sonography was given in the following order: second-trimester to third-trimester sonography (target scan), first-trimester sonography (target scan), first-trimester sonography, or second-trimester to third-trimester sonography. Preterm deliveries with a specified GA were given precedence over those without, with the LMP estimated by subtracting 35 weeks from the outcome date for unspecified preterm deliveries [4,23]. The latest LMP was selected with the smallest GA estimated by abortion or sonography codes for abortions, and sonography and preterm codes for deliveries.

Second, in the absence of codes indicating GA or trimester, we estimated the LMP by subtracting 39 weeks, 28 weeks, and 10 weeks from the date of the pregnancy outcomes for delivery, stillbirth, and abortion, respectively (Supplementary Material 6-2). This method is referred to as “outcome-specific estimates” and is similar to the conventionally used fixed-week subtraction method [4].

Final adjustment of the LMP was conducted using a retry period and setting minimum and maximum clinically plausible GAs for each outcome. The retry period, which represents the duration clinically required for subsequent pregnancy episodes to start after pregnancy outcome, was applied when two successive pregnancy episodes overlapped [4] (Supplementary Material 7). As illustrated in Supplementary Material 8, if the estimated LMP was earlier than the date of the previous pregnancy outcome, the LMP of the subsequent pregnancy episode was adjusted to the date of the previous pregnancy outcome plus the retry period.

Episodes not within the minimum and maximum GAs for each outcome were either reclassified or excluded [6-23]. Stillbirth episodes at less than 20 weeks GA were reclassified as abortions through case review. Deliveries at less than 37 weeks were classified as preterm and deliveries at 37 weeks or more as full-term births. Any pregnancy episodes with estimated GAs exceeding 42 weeks and deliveries at less than 20 weeks were considered miscoded and excluded. Abortion was further categorized into induced abortions, defined as abortions with diagnosis codes or procedure codes for induced abortion, and spontaneous abortions, defined as abortions without induced abortion codes (Supplementary Material 1).

### Application of the pregnancy algorithm among patients with systemic lupus erythematosus

The pregnancy episodes of women of childbearing age (15-49 years) with SLE (KCD-7: M32.0, and rare intractable disease registration: V136) were identified from NHID from 2002 to 2018. We included pregnancy episodes after the index date of SLE diagnosis, and those with an LMP between 2005 and 2018 (Supplementary Material 9). A 3-year history period was examined to confirm the diagnosis of SLE before pregnancy. In drug utilization analysis, pregnancy episodes that started at least 1 year after the index date of SLE and had an LMP before 2018 were included to examine exposure during preconception and pregnancy. Episodes missing age, gender, and insurance information at the time of the LMP were excluded.

### Identification of pregnancy outcomes and gestational age estimation

The prevalence of live births (full-term and preterm births), stillbirths, and abortions (spontaneous and induced abortions) was assessed in the pregnancies of patients with SLE between 2005 and 2018. The mean estimated GA and proportion of methods for estimating LMP were calculated for each pregnancy outcome. Since the national health insurance in Korea expanded reimbursement for prenatal sonography in 2016 and extended coverage for preterm infant care starting in 2016, the corresponding changes were made to the sonography procedure codes and preterm delivery diagnosis codes from that year onwards [24,25] (Supplementary Materials 10-12). Therefore, subgroups of 2005-2015 and 2016-2017 were also assessed to detect the impact of the code changes implemented in 2016. The prevalence of pregnancy outcomes among patients with SLE, as determined by our algorithm, was compared with that reported in cohort studies, primarily utilizing institutional data, to assess external validity [21,26-31].

### Medication use during pregnancy among patients with systemic lupus erythematosus

The European Alliance of Associations for Rheumatology and American College of Rheumatology guideline for the management of pregnancy recommends avoiding cyclophosphamide (CYC), mycophenolate mofetil (MMF), and methotrexate (MTX) to prevent fetal loss or malformation during pregnancy [15,17]. It is recommended to avoid MTX and CYC before conception, and MMF must be discontinued at least 6 weeks before conception [17]. CYC is reserved for use only in the second and third trimesters in case of life-threatening disease [15,17].

The use of CYC/MMF/MTX drugs during preconception (every 3 months before the LMP) and pregnancy periods (each trimester) was examined. To ascertain drug exposure and reduce misclassification bias, exposure to CYC/MMF/MTX was defined as having received more than one (> 1) prescription during the relevant period, and a sensitivity analysis was conducted using an exposure definition of one or more (≥ 1) prescription [14]. Exposure to CYC/MMF/MTX was investigated based on pregnancy outcomes, and subgroup analysis was conducted for the periods of 2005-2015 and 2016-2017. The medication exposure window was defined based on our new algorithm to estimate the LMP and the conventional algorithm (using only outcome-specific estimates). All analyses were also applied to non-steroidal anti-inflammatory drugs (NSAIDs).

### Ethics statement

The study protocol for the analysis of de-identified patient data was approved by Hanyang University Bioethics Committee (IRB No. HYUH 2020-05-041). The requirement for informed consent was waived by the institutional review board.

## RESULTS

In total, 5,800 pregnancy episodes were identified from 2005 to 2018 among 3,513 women of childbearing age (15-49 years) with SLE by applying our algorithm to the NHID (Supplementary Material 9). Live birth, stillbirth, and abortion accounted for 67% (95% confidence interval [CI], 46 to 88), 2% (95% CI, 0 to 6), and 31% (95% CI, 17 to 45) of pregnancy episodes, respectively (Table 1). Preterm births constituted 16.7% of the total live births and induced abortions accounted for 10.4% of the total abortions. The percentages of preterm births and stillbirths were 11% and 2%, respectively. Spontaneous abortions accounted for 28% of all pregnancy episodes. When restricting the spontaneous abortion definition to O02-O06, the prevalence of spontaneous abortion was 26% (95% CI, 13 to 39) (Supplementary Material 13). The mean± standard deviation estimated GA was 37.8 ± 2.8 weeks for live birth, 27.0± 2.4 weeks for stillbirth, and 8.5± 2.8 weeks for abortion (Table 1).

The LMP estimation for full-term births was primarily based on outcome-specific estimates (92.3%) (Table 2). For preterm births, sonography procedure codes (54.7%) and preterm delivery diagnosis codes (37.9%) most frequently determined the estimates. The LMP for stillbirths was mostly estimated using outcome-specific estimates (90.2%). The LMP for spontaneous abortion was estimated using outcome-specific estimates (58.2%) and abortion procedure codes (33.7%), whereas the LMP for induced abortion was primarily estimated using abortion procedure codes (95.2%). In Korea, sonography codes for pregnant women were implemented in 2013 and have been used widely since 2016 with coverage expansion for pregnant women (Supplementary Materials 10 and 11). As a result, since 2016, most LMP estimates have been based on sonography codes (Supplementary Material 14). The mean GAs by year in 2005-2015 and 2016-2017 showed statistically significant differences for live birth and spontaneous abortion (p< 0.001, p= 0.005) (Supplementary Material 15). Additionally, a higher prevalence of preterm birth and abortion was observed in 2016-2017 than in 2005-2015 (Supplementary Material 16).

**Figure 2.**
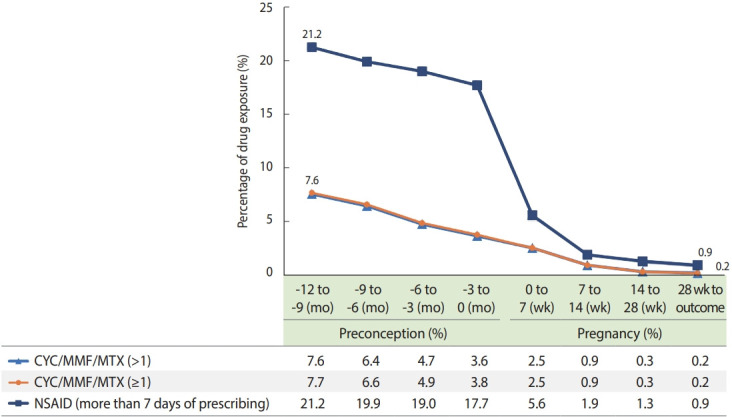
Cyclophosphamide (CYC)/mycophenolate mofetil (MMF)/methotrexate (MTX), and non-steroidal anti-inflammatory drugs (NSAID) use during preconception and pregnancy period.

The utilization patterns of CYC/MMF/MTX and NSAIDs before and during pregnancy were investigated in patients with SLE (Figure 2). The percentage of CYC/MMF/MTX use was similar between the two definitions (> 1 and ≥ 1 prescription). The use of CYC/MMF/MTX ( > 1) decreased from 7.6% at a year during preconception to 0.2% at the end of pregnancy. During -3 months to 0 months preconception, exposure to CYC/MMF/MTX occurred in 3.6% of cases and in 2.5% of cases at 0-7 weeks of pregnancy. NSAID use decreased from 21.2% a year during preconception to 0.9% at the end of pregnancy. A sharp decrease in NSAID use was observed between -3 months to 0 months preconception and 0-7 weeks of pregnancy. Greater exposure to CYC/MMF/MTX during -3 months to 0 months preconception was observed in cases of spontaneous abortion than in cases of full-term birth (8.2 vs. 1.4%), which is consistent with observations in the subgroups of 2005-2015 and 2016-2017 (Supplementary Material 17).

## DISCUSSION

We developed the present algorithm based on a previous algorithm and adapted it to a nationwide claims database in Korea by applying a hierarchy of pregnancy outcomes and incorporating preterm delivery, sonography, and abortion procedure codes to approximate the LMP. The current approach is the first to use the Korean administrative claims database to determine pregnancy outcomes and refine conventional outcome-specific methods for estimating the LMP.

This algorithm builds upon previous ones, particularly those developed by Matcho et al. [4] and Moll et al. [6], to improve the validity of pregnancy outcomes and the LMP using national administrative databases. Matcho et al. [4] and Moll et al. [6] demonstrated improved validity in LMP estimation compared to conventional outcome-specific approaches, which had previously reported 76.3% agreement rates of live birth with the clinical GA from discharged records within 1 week [23]. Matcho et al. [4] reported higher agreement for the LMP with reviewer evaluations than outcome-specific estimates (live birth: 91 vs. 88.1%), utilizing multiple data sources, including employer-based United States administrative health claims, Medicaid, private insurance claims, and United Kingdom-based electronic health records. Another study by Moll et al. [6] utilized a claims-electronic medical records dataset from the United States and reported improved LMP agreement with physician adjudication of electronic medical records for full-term (85.9%) and preterm (81.7%) births within 7 days. This improvement could be attributed to the application of screening tests, minimum and maximum pregnancy terms, and exclusion of episodes not adhering to clinical guidelines [4,6]. Likewise, our algorithm also incorporated screening tests, minimum and maximum pregnancy terms, and a clinically plausible period for the subsequent outcome and LMP.

Identifying preterm births from administrative claims databases is a known challenge in the literature, given the significant impact even a 1-week misclassification can have on pregnancy outcomes. Moll et al. [6] reported an agreement rate of 62.4% for preterm births with adjudicator-identified results, while revealing a much higher rate of 97.8% for full-term births. In another study using a British Columbia administrative database, Margulis et al. [23] reported positive predictive values (PPVs) ranging from 74% to 91% for the ICD-9/10 based definition of preterm status. In our study, the prevalence of preterm birth among patients with SLE was found to be 11%, which is lower than the range reported in institutional studies (13-40%) [26-30,32]. The underuse of preterm and sonography codes was observed before 2016 in the NHID, reflecting the expansion of reimbursement policy coverage in 2016 for preterm infant care and prenatal sonography [24,25]. Furthermore, preterm delivery diagnoses with a specified GA window were implemented in the Korean administrative claims database in 2016 (Supplementary Material 12). These changes in reimbursement policy contributed to underestimation of preterm birth prevalence before 2016.

From 2005 to 2015, underutilized preterm and sonography codes led to an underestimation of preterm births, while in 2016 and 2017, there was an observable shift towards overestimation (Supplementary Material 16). Interestingly, two institutional studies on SLE in Korea, conducted by Seo et al. [27] and Koh et al. [26], reported a higher prevalence of preterm birth rates (27.2 and 32.4%), respectively, than our estimates. These findings align with the prevalence estimated in our study after 2016, which was 33% for preterm birth <37 weeks and 21% for preterm birth < 34 weeks (Supplementary Material 16). As such, while the use of our algorithm requires caution, due to the absence of a validity study, we anticipate that our algorithm will achieve improved accuracy for the LMP, with the increasing use of preterm diagnosis and sonography codes specifying GA or trimesters.

We also observed a higher prevalence of spontaneous abortion (28%) among patients with SLE compared to the previously reported range (5-22%) [21,27-30]. A prior study, which used nationwide claims databases in Korea, reported a spontaneous abortion prevalence of 22% but utilized more restricted definition (O02-O06) compared to definition used in our study (O01-O08) [21]. Applying the same definition led to a slightly lower estimated prevalence of spontaneous abortion at 26% (Supplementary Material 13). Several factors may contribute to the higher prevalence observed in our study, including recent increase in maternal age in Korea and a longer follow-up period (2005-2017 vs. 2013-2015) [21,33].

Although a high prevalence of spontaneous abortion was observed in the present study, we implemented a hierarchical differentiation of various pregnancy outcomes, with abortion being the lowest category. Specifically, (1) abortion cases adjacent to delivery or stillbirth cases and (2) clinically implausible abortion cases based on both previous and subsequent episodes were excluded from abortion episodes in our final analysis. This approach is in line with the best-performing method identified in a previous validation study for non-live births using Medicaid in the Mass General Brigham Patient Registry, which showed adequate PPVs when excluding cases with adjacent codes for other pregnancy outcomes from the definition of abortion [32]. Further validation of abortion codes within the NHID is warranted due to differences in the healthcare system and coding practices.

In drug utilization analysis, CYC/MMF/MTX exposure was successfully reduced from 7.6% before conception to 0.2% during the third trimester. However, CYC/MMF/MTX was still prescribed in 3.6% of cases during -3 months to 0 months before conception. A gradual reduction in CYC/MMF/MTX was observed before conception, in contrast to the drastic decrease in utilization seen with NSAIDs at conception. This pattern may reflect the clinical patterns of discontinuing NSAIDs before the first trimester and suggests insufficient therapeutic interventions to discontinue CYC/MMF/MTX before conception in patients with SLE. Further studies are required to investigate patterns of CYC/MMF/MTX use during pregnancy in patients with SLE.

A key strength of our study is large dataset of pregnant women with SLE, which is particularly valuable due to the rarity of this population. In contrast to previously studies with 100-200 SLE pregnancy cases, our study benefits from increased statistical power [26,27,29,31,32]. Our pregnancy algorithm refined previous outcome-specific methods; however, it is important to emphasize that this algorithm is not a validation study using a database linked with electronic medical records or pregnancy registry. Acknowledging the algorithm’s limitations is essential, especially in the context of the Korean reimbursement system, where the widespread use of sonography procedures and preterm diagnosis codes began in 2016. Caution is needed when applying and interpreting data for different periods as well as estimating preterm births. The utilization of O60 codes, which continued until 2010, might have included preterm labor without delivery, potentially resulting in an overestimation of preterm births. Preterm births were predominantly identified through sonography, which may include cases categorized as preterm based on GA but not necessarily requiring clinical care for preterm infants. Furthermore, sonography codes might underestimate GA and be given priority over the LMP estimated by the preterm diagnosis, as our algorithm prioritizes the LMP with the lowest GA. To address these complexities, additional validation studies should investigate the hierarchy between sonography and preterm codes.

Misclassification bias and limited clinical data are inherent drawbacks of claims databases, particularly in capturing pregnancy episodes without healthcare utilization, potentially resulting in outcome underestimation. However, algorithms such as those from Matcho et al. [4] and Moll et al. [6], using hierarchical approaches with various pregnancy markers, have demonstrated improved validity compared to conventional methods.

In this study, we developed an advanced algorithm for determining pregnancy outcomes and estimating the LMP using an administrative claims database in Korea. The algorithm incorporates a hierarchical approach to mitigate miscoding or cross-coding, a clinically plausible gap for subsequent LMP and outcomes, and diagnoses and procedures with the trimester or GA. Further validation studies, including comparison with pregnancy registries or electronic health records, are needed [34].

## Figures and Tables

**Figure f3-epih-46-e2024012:**
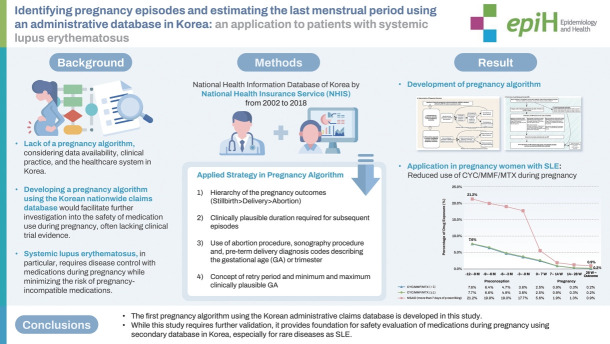


**Table 1.** Prevalence of pregnancy outcomes and gestational age among pregnancy episodes of women with systemic lupus erythematosus (2005-2018)

Pregnancy outcomes	Prevalence		Gestational age (wk)			
	n	% (95% CI)	Mean±SD	Median (Q1, Q3)	Min	Max
Live birth	3,871	67 (46, 88)	37.8±2.8	39 (38, 39)	20	42
Full-term	3,224	56 (36, 75)	38.9±0.5	39 (39, 39)	37	42
Preterm	647	11 (3, 20)	32.5±3.3	34 (30, 35)	20	36
Stillbirth	132	2 (0, 6)	27.0±2.4	28 (27, 28)	20	34
Abortion	1,797	31 (17, 45)	8.5±2.8	9 (6, 10)	3	20
Spontaneous	1,610	28 (14, 41)	8.4±2.2	9 (6, 10)	5	19
Induced	187	3 (0, 8)	9.6±5.8	10 (4, 14)	3	20

CI, confidence interval; SD, standard deviation; Min, minimum; Max, maximum.

**Table 2.** Algorithm applied to estimate the last menstrual period for pregnancy outcomes (2005-2018)

Algorithm	Total (n=5,800)	Full-term (n=3,224)	Preterm (n=647)	Stillbirth (n=132)	Spontaneous abortion (n=1,610)	Induced abortion (n=187)
Without abortion, sonography, preterm delivery claims						
Outcome-specific estimates	4,080 (70.3)	2,976 (92.3)	48 (7.4)	119 (90.2)	937 (58.2)	0 (0.0)
With abortion, sonography, preterm delivery claims						
Abortion procedure codes	720 (12.4)	0 (0.0)	0 (0.0)	0 (0.0)	542 (33.7)	178 (95.2)
Sonography procedure codes^1^	755 (13.0)	248 (7.7)	354 (54.7)	13 (9.8)	131 (8.1)	9 (4.8)
Sonography code during the first trimester	131 (2.3)	3 (0.1)	3 (0.5)	1 (0.8)	116 (7.2)	8 (4.3)
Sonography code during the second and third trimesters	185 (3.2)	6 (0.2)	177 (27.4)	2 (1.5)	0 (0.0)	0 (0.0)
Sonography code during the first trimester-TS	27 (0.5)	12 (0.4)	0 (0.0)	1 (0.8)	13 (0.8)	1 (0.5)
Sonography code during the second and third trimesters-TS	412 (7.1)	227 (7.0)	174 (26.9)	9 (6.8)	2 (0.1)	0 (0.0)
Preterm delivery diagnosis codes^2^	245 (4.2)	0 (0.0)	245 (37.9)	0 (0.0)	0 (0.0)	0 (0.0)
Preterm code with specified period	28 (0.5)	0 (0.0)	28 (4.3)	0 (0.0)	0 (0.0)	0 (0.0)
Preterm code without specified period	217 (3.7)	0 (0.0)	217 (33.5)	0 (0.0)	0 (0.0)	0 (0.0)

Values are presented as number (%). TS, target scan.

## Data Availability

The data underlying this article cannot be shared publicly for the privacy of individuals in the claims database. The data will be shared on reasonable request to the corresponding author.

## Supplementary materials

Supplementary Material 1. Definition of pregnancy outcomes

Supplementary Material 2. Distribution of procedure codes and diagnosis codes for delivery

Supplementary Material 3. Distribution of procedure codes and diagnosis codes for abortion

Supplementary Material 4. A sequential illustration of pregnancy episode determination

Supplementary Material 5. Clinically plausible duration required for subsequent episode

Supplementary Material 6-1. Estimation of LMP with presence of abortion, sonography, and preterm codes

Supplementary Material 6-2. Estimation of LMP without presence of abortion, sonography, and preterm codes (Outcome-specific estimates)

Supplementary Material 7. Retry period

Supplementary Material 8. Illustration of LMP adjustment by retry period

Supplementary Material 9. Pregnancy episodes of patients with SLE

Supplementary Material 10. Sonography 1^st^ trimester procedure codes (2005–2018) from the total dataset by claims records

Supplementary Material 11. Sonography 2^nd^, 3^rd^ trimester procedure codes (2005–2018) from the total dataset by claims records

Supplementary Material 12. Pre-term delivery diagnosis codes (2005–2018) from the total dataset by claims records

Supplementary Material 13. Prevalence of pregnancy outcomes among systemic lupus erythematosus (SLE) women by restricting abortion definition to O02–O06 (2005–2018)

Supplementary Material 14. Applied algorithm to estimate LMP from 2005 to 2018

Supplementary Material 15. Average gestational age (GA) in weeks from 2005–2015, and 2016–2017

Supplementary Material 16. Prevalence of pregnancy outcomes among systemic lupus erythematosus (SLE) women by 2005–2015 and 2016–2017.

Supplementary Material 17. Exposure of CYC/MMF/MTX during 3 months before conception and 1st trimester (medications for long-term use)
