# Supplementary material for: Identifying pregnancy episodes and estimating the last menstrual period using an administrative database in Korea: an application to patients with systemic lupus erythematosus
Source: Epidemiol Health. 2023 Dec 19;46:e2024012. doi: 10.4178/epih.e2024012 (PMC11040213; doi:10.4178/epih.e2024012)
Supplement: Supplementary Material 1. — Definition of pregnancy outcomes [file epih-46-e2024012-Supplementary-1.docx]

**Supplementary Material 1** Definition of pregnancy outcomes

| **Pregnancy Outcomes** | | **Definition** |
| --- | --- | --- |
| Stillbirth  (KCD-7 code only) | | Z371, Z373, Z374, Z376, Z377, O364 |
| Delivery  (Procedure code only) | | R313, R3131, R3133, R3136, R3138, R314, R3141, R3143, R3146, R3148, R435, R4351, R4353, R4356, R4358, R436, R4361, R4362, R438, R4380, R450, R4507, R4508, R4509, R451, R4510, R4514, R4516, R4517, R4518, R4519, R4520, R4522, R500, R5001, R5002, RA31, RA311, RA312, RA313, RA314, RA315, RA316, RA317, RA318, RA36, RA361, RA362, RA38, RA380, RA43, RA431, RA432, RA433, RA434 |
| Abortion  (KCD-7code only) | | O01, O02, O03, O04, O05, O06, O07, O08 |
| Induced abortion (KCD-7 code or procedure code) | (KCD-7 code) O04  (Procedure code) R4452, R4456, R4457, R4458, R4459 |  |
| Spontaneous abortion | All abortion episode without codes indicative of induced abortion |  |
