# Supplementary figures and images for: Identifying pregnancy episodes and estimating the last menstrual period using an administrative database in Korea: an application to patients with systemic lupus erythematosus
Source: Epidemiol Health. 2023 Dec 19;46:e2024012. doi: 10.4178/epih.e2024012 (PMC11040213; doi:10.4178/epih.e2024012)

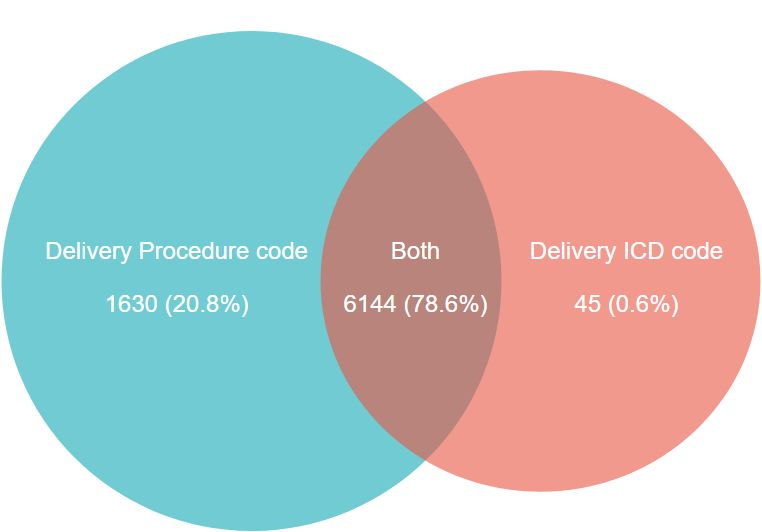


Supplementary Material 2 Distribution of procedure codes and diagnosis codes for delivery

Supplement: Supplementary Material 2. — Distribution of procedure codes and diagnosis codes for delivery [file epih-46-e2024012-Supplementary-2.docx]

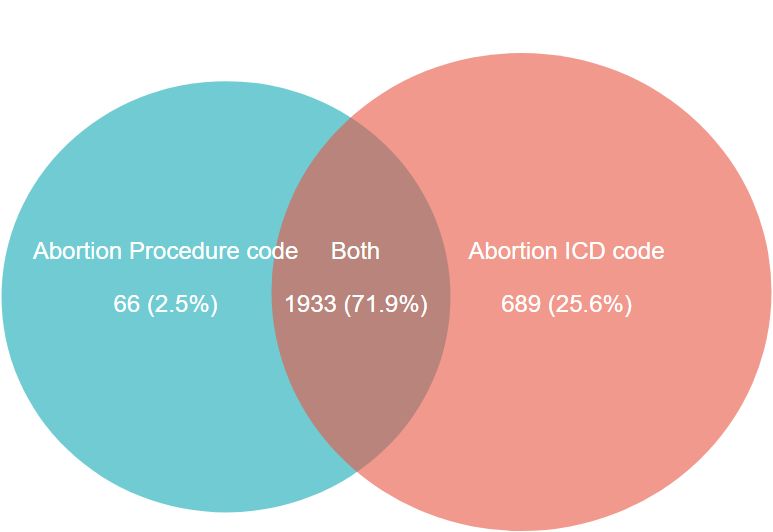


**Supplementary Material 3** Distribution of procedure codes and diagnosis codes for abortion

Supplement: Supplementary Material 3. — Distribution of procedure codes and diagnosis codes for abortion [file epih-46-e2024012-Supplementary-3.docx]

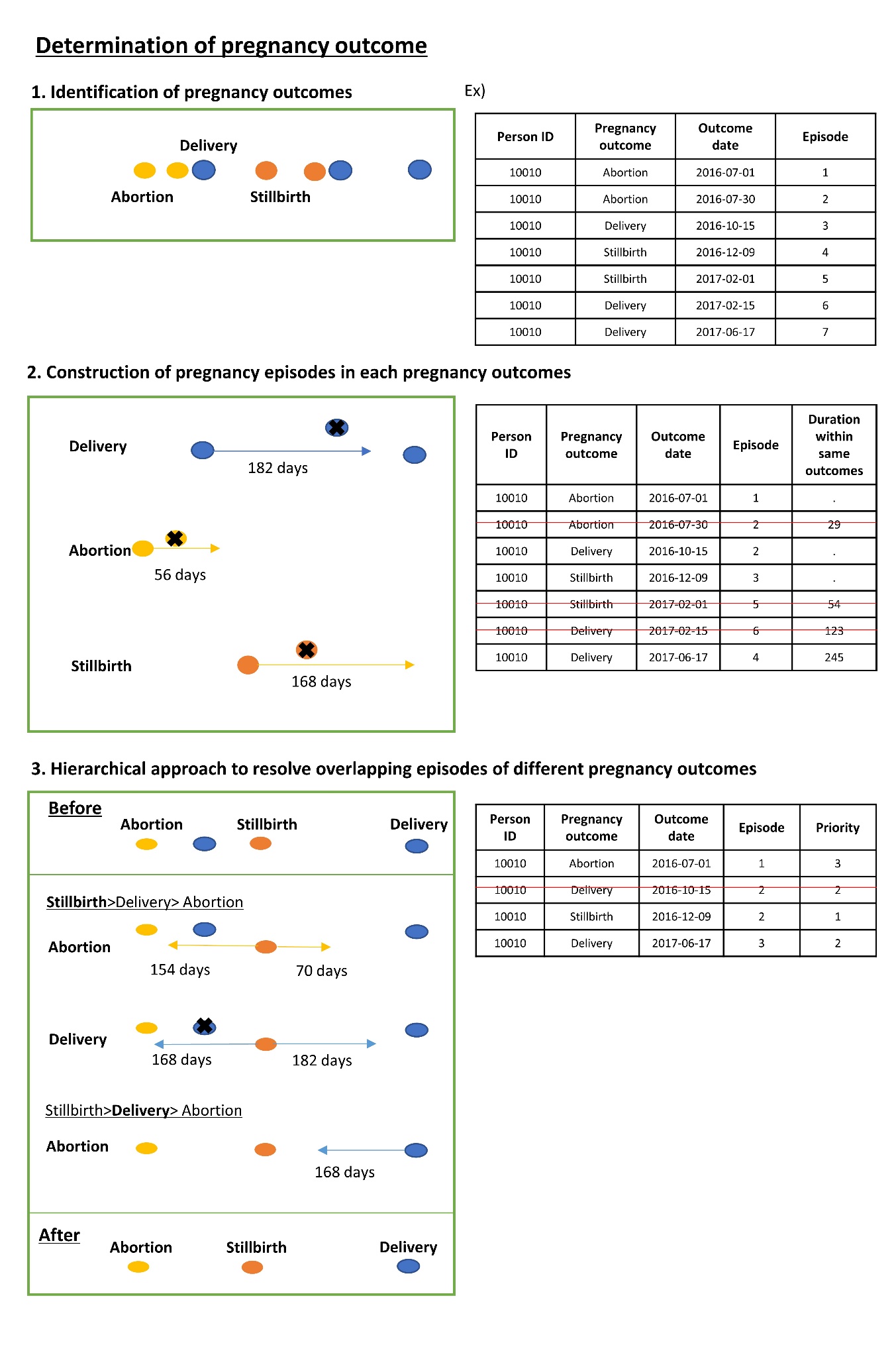
 Supplementary Material 4 A sequential illustration of pregnancy episode determination

Supplement: Supplementary Material 4. — A sequential illustration of pregnancy episode determination [file epih-46-e2024012-Supplementary-4.docx]

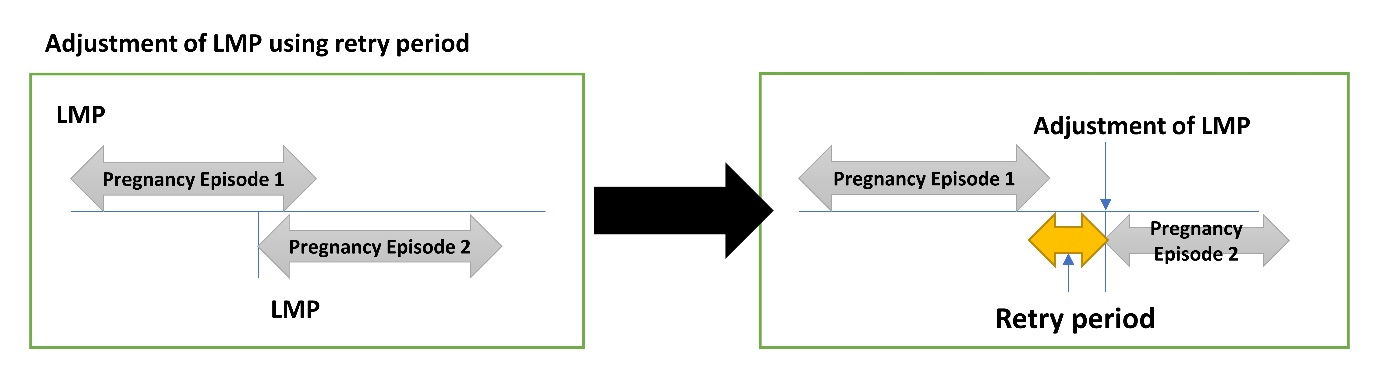


Supplementary Material 8 Illustration of LMP adjustment by retry period

Supplement: Supplementary Material 8. — Illustration of LMP adjustment by retry period [file epih-46-e2024012-Supplementary-8.docx]

**
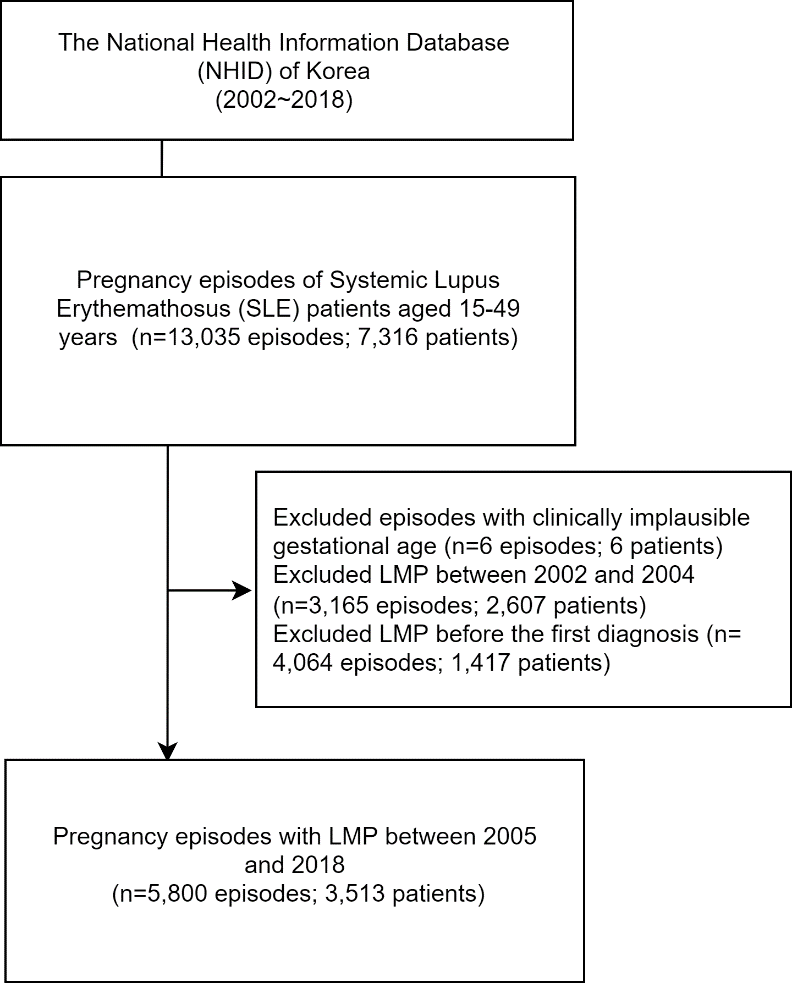
**

**Supplementary Material 9** Pregnancy episodes of patients with SLE

Supplement: Supplementary Material 9. — Pregnancy episodes of patients with SLE [file epih-46-e2024012-Supplementary-9.docx]

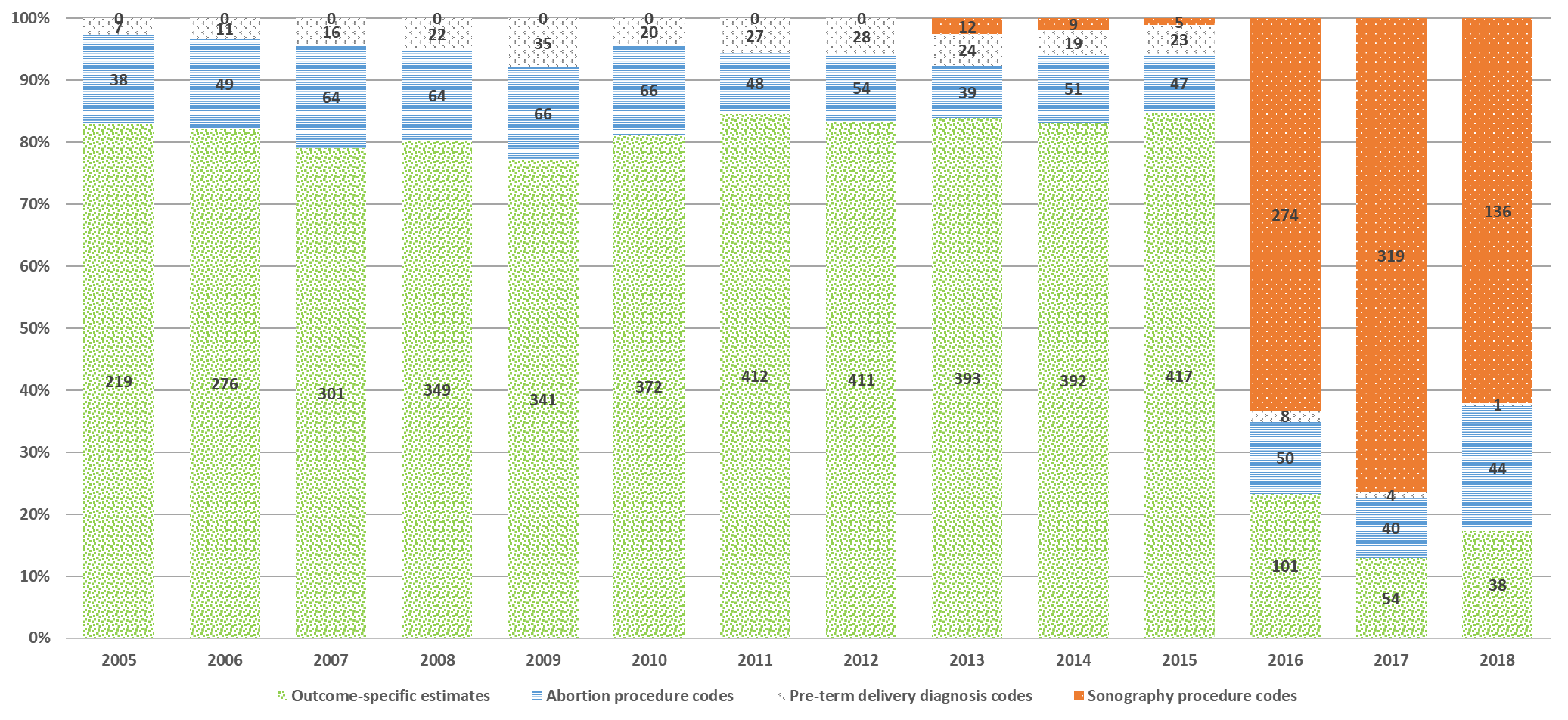


Supplementary Material 14 Applied algorithm to estimate LMP from 2005 to 2018

Supplement: Supplementary Material 14. — Applied algorithm to estimate LMP from 2005 to 2018 [file epih-46-e2024012-Supplementary-14.docx]
