# Supplementary material for: Identifying pregnancy episodes and estimating the last menstrual period using an administrative database in Korea: an application to patients with systemic lupus erythematosus
Source: Epidemiol Health. 2023 Dec 19;46:e2024012. doi: 10.4178/epih.e2024012 (PMC11040213; doi:10.4178/epih.e2024012)
Supplement: Supplementary Material 5. — Clinically plausible duration required for subsequent episode [file epih-46-e2024012-Supplementary-5.docx]

**Supplementary Material 5** Clinically plausible duration required for subsequent episode

| **Pregnancy Episode** | **Subsequent Pregnancy Episode** | **Minimum Duration (Days)** |
| --- | --- | --- |
| Delivery | Delivery | 182 |
| Delivery | Stillbirth | 168 |
| Delivery | Abortion | 70 |
| Stillbirth | Delivery | 182 |
| Stillbirth | Stillbirth | 168 |
| Stillbirth | Abortion | 70 |
| Abortion | Delivery | 168 |
| Abortion | Stillbirth | 154 |
| Abortion | Abortion | 56 |
