# Supplementary material for: Identifying pregnancy episodes and estimating the last menstrual period using an administrative database in Korea: an application to patients with systemic lupus erythematosus
Source: Epidemiol Health. 2023 Dec 19;46:e2024012. doi: 10.4178/epih.e2024012 (PMC11040213; doi:10.4178/epih.e2024012)
Supplement: Supplementary Material 6-1. — Estimation of LMP with presence of abortion, sonography, and preterm codes [file epih-46-e2024012-Supplementary-6-1.docx]

**Supplementary Material 6-1** Estimation of LMP with presence of abortion, sonography, and preterm codes

| **Code** | **Procedure code/KCD-7 code** | **Definition** | **Midpoint**  **(weeks)** | **Estimation of LMP** |
| --- | --- | --- | --- | --- |
| Abortion^e^ | R4452 | Induced abortion < 8 weeks | 4 | LMP=Date of pregnancy outcome-4*7+1 |
|  | R4441 | Miscarriage, dilation & curettage <12 weeks | 6 | LMP=Date of pregnancy outcome-6*7+1 |
|  | R4456 | Induced abortion 8–12 weeks | 10 | LMP=Date of pregnancy outcome-10*7+1 |
|  | R4457 | Induced abortion 12–16 weeks | 14 | LMP=Date of pregnancy outcome-14*7+1 |
|  | R4442 | Miscarriage, dilation & curettage ≥ 12 weeks | 16 | LMP=Date of pregnancy outcome-16*7+1 |
|  | R4458 | Induced abortion 16–20 weeks | 18 | LMP=Date of pregnancy outcome-18*7+1 |
|  | R4459 | Induced abortion ≥ 20 weeks | 20 | LMP=Date of pregnancy outcome-20*7+2 |
| Sonography^f^ | Sonography 1st trimester (0–14 weeks) | | 7 | LMP=Date of pregnancy outcome-7*7+1 |
|  | E9471^a^ | 1st trimester - General |  |  |
|  | EB511^b^ | 1st trimester - General |  |  |
|  | EB512 ^b^ | 1st trimester - General - Pregnancy confirmation |  |  |
|  | Sonography 1st trimester –  Target Scan (11–14 weeks) | | 12.5 | LMP=Date of pregnancy outcome-12.5*7+1 |
|  | E9473 ^a^ | 1st trimester - Target scan |  |  |
|  | EB513 ^b^ | 1st trimester - Target scan |  |  |
|  | EB514 ^b^ | 1st trimester - Target scan - Fetal malformation |  |  |
|  | Sonography 2nd and 3rd trimester (14–42 weeks) | | 28 | LMP=Date of pregnancy outcome-28*7+1 |
|  | E9472^a^ | 2nd and 3rd trimester - General |  |  |
|  | EB515^b^ | 2nd and 3rd trimester - General |  |  |
|  | EB516^b^ | 2nd and 3rd trimester - High-risk pregnancy |  |  |
|  | Sonography 2nd and 3rd trimester –  Target Scan (20–22 weeks) | | 21 | LMP=Date of pregnancy outcome-21*7+1 |
|  | E9474^a^ | 2nd and 3rd trimester - Target scan |  |  |
|  | EB517^b^ | 2nd and 3rd trimester - Target scan |  |  |
|  | EB518^b^ | 2nd and 3rd trimester - Target scan - Fetal malformation |  |  |
| Pre-term delivery, specified gestational age^e^ | O6010^b^ | Pre-term delivery with pre-term labor (<22 weeks) | 21 | LMP=Date of pregnancy outcome-21*7+1 |
|  | O6030^b^ | Pre-term delivery without labor (<22 weeks) | 21 | LMP=Date of pregnancy outcome-21*7+1 |
|  | O6011^b^ | Pre-term delivery with pre-term labor (22–34 weeks) | 28 | LMP=Date of pregnancy outcome-28*7+1 |
|  | O6031^b^ | Pre-term delivery without labor (22–34 weeks) | 28 | LMP=Date of pregnancy outcome-28*7+1 |
|  | O6012^b^ | Pre-term delivery with pre-term labor (≥ 34 weeks) | 35.5 | LMP=Date of pregnancy outcome-35.5*7+1 |
|  | O6032^b^ | Pre-term delivery without labor (≥ 34 weeks) | 35.5 | LMP=Date of pregnancy outcome-35.5*7+1 |
| Pre-term delivery, not specified^e^ | O60^c^ | Pre-term labor or pre-term delivery | 35 | LMP=Date of pregnancy outcome-35*7+1 |
|  | O601^d^ | Pre-term delivery with pre-term labor | 35 | LMP=Date of pregnancy outcome-35*7+1 |
|  | O6013^b^ | Pre-term delivery with pre-term labor, unknown gestational age | 35 | LMP=Date of pregnancy outcome-35*7+1 |
|  | O603^d^ | Pre-term delivery without labor | 35 | LMP=Date of pregnancy outcome-35*7+1 |

^a^ Codes utilized from 2013 to 2015 ^b^ Codes utilized from 2016 ^c^ Codes utilized from 2002 ^d^ Codes utilized from 2008 ^e^Abortion procedure and pre-term delivery diagnosis codes were investigated within 12 weeks prior to the date of the pregnancy outcome. ^f^Sonography procedure codes were identified based on the earliest plausible LMP using first-trimester sonography codes, with 13 and 35 weeks before the dates of abortion and delivery/stillbirth, respectively.
