# Supplementary material for: Identifying pregnancy episodes and estimating the last menstrual period using an administrative database in Korea: an application to patients with systemic lupus erythematosus
Source: Epidemiol Health. 2023 Dec 19;46:e2024012. doi: 10.4178/epih.e2024012 (PMC11040213; doi:10.4178/epih.e2024012)
Supplement: Supplementary Material 6-2. — Estimation of LMP without presence of abortion, sonography, and preterm codes (Outcome-specific estimates) [file epih-46-e2024012-Supplementary-6-2.docx]

**Supplementary Material 6-2** Estimation of LMP without presence of abortion, sonography, and preterm codes (Outcome-specific estimates)

| **Pregnancy Outcome** | **Weeks** | **Estimation of LMP** |
| --- | --- | --- |
| Delivery | 39 | LMP=Date of pregnancy outcome-39*7+1 |
| Stillbirth | 28 | LMP=Date of pregnancy outcome-28*7+1 |
| Abortion | 10 | LMP=Date of pregnancy outcome-10*7+1 |
