# Supplementary material for: Identifying pregnancy episodes and estimating the last menstrual period using an administrative database in Korea: an application to patients with systemic lupus erythematosus
Source: Epidemiol Health. 2023 Dec 19;46:e2024012. doi: 10.4178/epih.e2024012 (PMC11040213; doi:10.4178/epih.e2024012)
Supplement: Supplementary Material 7. — Retry period [file epih-46-e2024012-Supplementary-7.docx]

**Supplementary Material 7** Retry period

| **Pregnancy episode** | **Retry period: days needed for initiation of successive pregnancy** |
| --- | --- |
| Delivery | 28 |
| Stillbirth | 28 |
| Abortion | 14 |
