# Supplementary material for: Identifying pregnancy episodes and estimating the last menstrual period using an administrative database in Korea: an application to patients with systemic lupus erythematosus
Source: Epidemiol Health. 2023 Dec 19;46:e2024012. doi: 10.4178/epih.e2024012 (PMC11040213; doi:10.4178/epih.e2024012)
Supplement: Supplementary Material 10. — Sonography 1st trimester procedure codes (2005–2018) from the total dataset by claims records [file epih-46-e2024012-Supplementary-10.docx]

**Supplementary Material 10** Sonography 1^st^ trimester procedure codes (2005–2018) from the total dataset by claims records

| **Year** | **1st trimester** | | | | | | | | | | | |
| --- | --- | --- | --- | --- | --- | --- | --- | --- | --- | --- | --- | --- |
|  | **E9471** | **E9473** | **EB511** | **EB511001** | **EB511010** | **EB511011** | **EB512** | **EB512010** | **EB513** | **EB513010** | **EB514** | **EB514010** |
| 2005 | - | - | - | - | - | - | - | - | - | - | - | - |
| 2006 | - | - | - | - | - | - | - | - | - | - | - | - |
| 2007 | - | - | - | - | - | - | - | - | - | - | - | - |
| 2008 | - | - | - | - | - | - | - | - | - | - | - | - |
| 2009 | - | - | - | - | - | - | - | - | - | - | - | - |
| 2010 | - | - | - | - | - | - | - | - | - | - | - | - |
| 2011 | - | - | - | - | - | - | - | - | - | - | - | - |
| 2012 | - | - | - | - | - | - | - | - | - | - | - | - |
| 2013 | - | - | - | - | - | - | - | - | - | - | - | - |
| 2014 | 14 | - | - | - | - | - | - | - | - | - | - | - |
| 2015 | 11 | 1 | - | - | - | - | - | - | - | - | - | - |
| 2016 | 2 | - | 189 | 4 | 68 | - | 43 | 6 | 50 | 37 | - | - |
| 2017 | - | - | 805 | 42 | 255 | 6 | 135 | 7 | 211 | 125 | 5 | 2 |
| 2018 | - | - | 882 | 26 | 152 | 7 | 117 | 4 | 213 | 213 | 3 | 2 |
