# Supplementary material for: Identifying pregnancy episodes and estimating the last menstrual period using an administrative database in Korea: an application to patients with systemic lupus erythematosus
Source: Epidemiol Health. 2023 Dec 19;46:e2024012. doi: 10.4178/epih.e2024012 (PMC11040213; doi:10.4178/epih.e2024012)
Supplement: Supplementary Material 11. — Sonography 2nd, 3rd trimester procedure codes (2005–2018) from the total dataset by claims records [file epih-46-e2024012-Supplementary-11.docx]

**Supplementary Material 11** Sonography 2^nd^, 3rd trimester procedure codes (2005–2018) from the total dataset by claims records

| **Year** | **2nd, 3rd trimester** | | | | | | | | | | | | |
| --- | --- | --- | --- | --- | --- | --- | --- | --- | --- | --- | --- | --- | --- |
|  | **E9472** | **E9474** | **E9472001** | **EB515** | **EB515001** | **EB515010** | **EB515011** | **EB516** | **EB516010** | **EB517** | **EB17010** | **EB518** | **EB51810** |
| 2005 | - | - | - | - | - | - | - | - | - | - | - | - | - |
| 2006 | - | - | - | - | - | - | - | - | - | - | - | - | - |
| 2007 | - | - | - | - | - | - | - | - | - | - | - | - | - |
| 2008 | - | - | - | - | - | - | - | - | - | - | - | - | - |
| 2009 | - | - | - | - | - | - | - | - | - | - | - | - | - |
| 2010 | - | - | - | - | - | - | - | - | - | - | - | - | - |
| 2011 | - | - | - | - | - | - | - | - | - | - | - | - | - |
| 2012 | - | - | - | - | - | - | - | - | - | - | - | - | - |
| 2013 | 5 | - | 1 | - | - | - | - | - | - | - | - | - | - |
| 2014 | 7 | 1 | - | - | - | - | - | - | - | - | - | - | - |
| 2015 | 9 | 2 | - | - | - | - | - | - | - | - | - | - | - |
| 2016 | 3 | 2 | - | 251 | 10 | 110 | 3 | 21 | 15 | 54 | 41 | 4 | 3 |
| 2017 | - | - | - | 927 | 99 | 390 | 26 | 97 | 54 | 182 | 154 | 4 | 12 |
| 2018 | - | - | - | 892 | 96 | 300 | 21 | 90 | 33 | 171 | 127 | 3 | 6 |
