# Supplementary material for: Identifying pregnancy episodes and estimating the last menstrual period using an administrative database in Korea: an application to patients with systemic lupus erythematosus
Source: Epidemiol Health. 2023 Dec 19;46:e2024012. doi: 10.4178/epih.e2024012 (PMC11040213; doi:10.4178/epih.e2024012)
Supplement: Supplementary Material 12. — Pre-term delivery diagnosis codes (2005–2018) from the total dataset by claims records [file epih-46-e2024012-Supplementary-12.docx]

**Supplementary Material 12** Pre-term delivery diagnosis codes (2005–2018) from the total dataset by claims records

| **Year** | **Pre-term Code** | | | | | | | | | | | | | | | |
| --- | --- | --- | --- | --- | --- | --- | --- | --- | --- | --- | --- | --- | --- | --- | --- | --- |
|  | **O60** | **O600** | **O6000** | **O6001** | **O6002** | **O6009** | **O601** | **O6010** | **O6011** | **O6012** | **O6019** | **O602** | **O6022** | **O603** | **O6031** | **O6032** |
| 2005 | 38 | - | - | - | - | - | - | - | - | - | - | - | - | - | - | - |
| 2006 | 30 | - | - | - | - | - | - | - | - | - | - | - | - | - | - | - |
| 2007 | 44 | - | - | - | - | - | - | - | - | - | - | - | - | - | - | - |
| 2008 | 4 | 32 | - | - | - | - | 31 | - | - | - | - | 1 | - | - | - | - |
| 2009 | 1 | 56 | - | - | - | - | 44 | - | - | - | - | 2 | - | - | - | - |
| 2010 | 1 | 82 | - | - | - | - | 56 | - | - | - | - | - | - | - | - | - |
| 2011 | - | 87 | - | - | - | - | 37 | - | - | - | - | 2 | - | 5 | - | - |
| 2012 | - | 123 | - | - | - | - | 44 | - | - | - | - | 3 | - | 15 | - | - |
| 2013 | - | 133 | - | - | - | - | 45 | - | - | - | - | 3 | - | 12 | - | - |
| 2014 | - | 178 | - | - | - | - | 40 | - | - | - | - | 1 | - | 13 | - | - |
| 2015 | - | 168 | - | - | - | - | 32 | - | - | - | - | 1 | - | 13 | - | - |
| 2016 | - | 1 | 17 | 82 | 40 | 36 | - | 2 | 3 | 15 | 1 | 1 | 3 | - | 12 | 5 |
| 2017 | - | 2 | 25 | 91 | 38 | 19 | - | - | 2 | 18 | 2 | - | - | - | 1 | 5 |
| 2018 | - | 2 | 22 | 112 | 31 | 14 | - | - | 14 | 12 | - | - | - | - | 2 | 1 |
