# Supplementary material for: Identifying pregnancy episodes and estimating the last menstrual period using an administrative database in Korea: an application to patients with systemic lupus erythematosus
Source: Epidemiol Health. 2023 Dec 19;46:e2024012. doi: 10.4178/epih.e2024012 (PMC11040213; doi:10.4178/epih.e2024012)
Supplement: Supplementary Material 13. — Prevalence of pregnancy outcomes among systemic lupus erythematosus (SLE) women by restricting abortion definition to O02–O06 (2005–2018) [file epih-46-e2024012-Supplementary-13.docx]

**Supplementary Material 13** Prevalence of pregnancy outcomes among systemic lupus erythematosus (SLE) women by restricting abortion definition to O02–O06 (2005–2018)

| **Prevalence of pregnancy outcomes** | **N** | **% (95%CI)** |
| --- | --- | --- |
| Total pregnancy episodes | 5,669 | 100% (74–100%) |
| Live birth | 3,871 | 68% (47–90%) |
| Full-term birth | 3,224 | 57% (36–76%) |
| Pre-term birth | 647 | 11% (3–20%) |
| Stillbirth | 132 | 2% (0-6%) |
| Abortion | 1,666 | 29% (15–43%) |
| Spontaneous abortion | 1,480 | 26% (13–39%) |
| Induced abortion | 186 | 3% (0–8%) |

CI: confidence interval
