# Supplementary material for: Identifying pregnancy episodes and estimating the last menstrual period using an administrative database in Korea: an application to patients with systemic lupus erythematosus
Source: Epidemiol Health. 2023 Dec 19;46:e2024012. doi: 10.4178/epih.e2024012 (PMC11040213; doi:10.4178/epih.e2024012)
Supplement: Supplementary Material 15. — Average gestational age (GA) in weeks from 2005–2015, and 2016–2017 [file epih-46-e2024012-Supplementary-15.docx]

**Supplementary Material 15** Average gestational age (GA) in weeks from 2005–2015, and 2016–2017

| **Pregnancy outcomes** | **2005**–**2015** | **2016**–**2017** | **Difference in GA** | **p-value** |
| --- | --- | --- | --- | --- |
| Livebirth | 38.4 (1.9) | 34.7 (4.2) | 3.7 | <0.001 |
| Full-term birth | 38.9 (0.3) | 38.5 (1.2) | 0.4 | <0.001 |
| Pre-term birth | 33.4 (3.2) | 31.7 (3.2) | 1.7 | <0.001 |
| Stillbirth | 27.2 (2.0) | 26.3 (3.6) | 0.9 | 0.108 |
| Abortion | 8.6 (2.9) | 8.2 (2.4) | 0.4 | 0.006 |
| Spontaneous abortion | 8.5 (2.2) | 8.2 (2.2) | 0.3 | 0.005 |
| Induced abortion | 9.6 (5.9) | 9.4 (4.8) | 0.2 | 0.915 |
