# Supplementary material for: Identifying pregnancy episodes and estimating the last menstrual period using an administrative database in Korea: an application to patients with systemic lupus erythematosus
Source: Epidemiol Health. 2023 Dec 19;46:e2024012. doi: 10.4178/epih.e2024012 (PMC11040213; doi:10.4178/epih.e2024012)
Supplement: Supplementary Material 16. — Prevalence of pregnancy outcomes among systemic lupus erythematosus (SLE) women by 2005–2015 and 2016–2017. [file epih-46-e2024012-Supplementary-16.docx]

**Supplementary Material 16** Prevalence of pregnancy outcomes among systemic lupus erythematosus (SLE) women by 2005–2015 and 2016–2017.

| **Pregnancy Outcome** | **2005**–**2015** | | **2016**–**2017** | |
| --- | --- | --- | --- | --- |
|  | **N** | **%** | **N** | **%** |
| Livebirth | 3,256 | 69% | 615 | 58% |
| Pre-term birth (<37 weeks) | 294 | 6% | 353 | 33% |
| Pre-term birth (<34 weeks) | 66 | 1% | 221 | 21% |
| Stillbirth | 112 | 2% | 20 | 2% |
| Abortion | 1,363 | 29% | 434 | 41% |
| Total pregnancy case | 4,731 | 100% | 1,069 | 100% |
