# Supplementary material for: Identifying pregnancy episodes and estimating the last menstrual period using an administrative database in Korea: an application to patients with systemic lupus erythematosus
Source: Epidemiol Health. 2023 Dec 19;46:e2024012. doi: 10.4178/epih.e2024012 (PMC11040213; doi:10.4178/epih.e2024012)
Supplement: Supplementary Material 17. — Exposure of CYC/MMF/MTX during 3 months before conception and 1st trimester (medications for long-term use) [file epih-46-e2024012-Supplementary-17.docx]

**Supplementary Material 17** Exposure of CYC/MMF/MTX during 3 months before conception and 1st trimester (medications for long-term use)

| **CYC/MMF/MTX** | **Full-term**  **birth** | | **Pre-term birth** | | **Stillbirth** | | **Spontaneous abortion** | | **Induced abortion** | | **p-value** |
| --- | --- | --- | --- | --- | --- | --- | --- | --- | --- | --- | --- |
|  | **N** | **%** | **N** | **%** | **N** | **%** | **N** | **%** | **N** | **%** |  |
| **Our algorithm to estimate LMP** | | | | | | | | | | | |
| Exposed during 0–3 months before LMP | 41 | 1.4% | 17 | 3.1% | 2 | 1.7% | 111 | 8.2% | 13 | 8.6% | <0.001 |
| Exposed during 1st trimester | 25 | 0.9% | 8 | 1.4% | 3 | 2.6% | 92 | 6.8% | 12 | 7.9% | <0.001 |
| Total episodes | 2,871 |  | 555 |  | 116 |  | 1,350 |  | 152 |  |  |
| 2005~2015 |  |  |  |  |  |  |  |  |  |  |  |
| Exposed during 0–3 months before LMP | 39 | 1.5% | 12 | 4.5% | 2 | 2.0% | 89 | 8.2% | 12 | 8.5% | <0.001 |
| Exposed during 1st trimester | 24 | 0.9% | 6 | 2.3% | 3 | 3.0% | 75 | 6.9% | 11 | 7.8% | <0.001 |
| Total episodes | 2,663 |  | 266 |  | 99 |  | 1,081 |  | 141 |  |  |
| 2016~2017 |  |  |  |  |  |  |  |  |  |  |  |
| Exposed during 0–3 months before LMP | 2 | 1.0% | 5 | 1.7% | 0 | 0.0% | 22 | 8.2% | 1 | 9.1% | <0.001 |
| Exposed during 1st trimester | 1 | 0.5% | 2 | 0.7% | 0 | 0.0% | 17 | 6.3% | 1 | 9.1% | <0.001 |
| Total episodes | 208 |  | 289 |  | 17 |  | 269 |  | 11 |  |  |
| **Previous estimation method (outcome-specific estimates only) for LMP** | | | | | | | | | | | |
| Exposed during 0–3 months before LMP | 42 | 1.5% | 24 | 4.3% | 2 | 1.7% | 120 | 8.9% | 11 | 7.2% | <0.001 |
| Exposed during 1st trimester | 27 | 0.9% | 11 | 2.0% | 3 | 2.6% | 95 | 7.0% | 12 | 7.9% | <0.001 |
| Total episodes | 2,871 |  | 555 |  | 116 |  | 1,350 |  | 152 |  |  |
